# Meta-analysis of the incidence and risks of interstitial lung disease and QTc prolongation in non-small-cell lung cancer patients treated with ALK inhibitors

**DOI:** 10.18632/oncotarget.18283

**Published:** 2017-05-29

**Authors:** Liping Lin, Juanjuan Zhao, Ning Kong, Yan He, Jiazhu Hu, Fuxi Huang, Jianjun Han, Xiaolong Cao

**Affiliations:** ^1^ Department of Oncology, Panyu Central Hospital, Guangzhou, 511400, China; ^2^ School of Nursing, Sun Yat-sen University, Guangzhou, 510000, China; ^3^ Department of Ophthalmology, Panyu Central Hospital, Guangzhou, 511400, China; ^4^ Cancer Institute of Panyu, Guangzhou, 511400, China

**Keywords:** ALK-TKIs, interstitial lung disease, QTc prolongation, non-small-cell lung cancer, meta-analysis

## Abstract

**Background:**

To conduct a systematic review and meta-analysis to assess the overall incidence and risk of interstitial lung disease (ILD) and QTc prolongation associated with anaplastic lymphoma kinase (ALK)-tyrosine kinase inhibitors (-TKIs) in non-small-cell lung cancer (NSCLC) patients.

**Results:**

A total of 1,770 patients from 8 clinical trials were included. The incidences of high-grade ILD and QTc prolongation was 2.5% (95% CI 1.7-3.6%), and 2.8% (95% CI 1.8-4.3%), respectively. Meta-analysis demonstrated that the use of ALK-TKIs in NSCLC patients significantly increased the risk of developing high-grade ILD (Peto OR, 3.27, 95%CI: 1.18–9.08, *p* = 0.023) and QTc prolongation (Peto OR 7.51, 95% CI, 2.16–26.15; *p* = 0.002) in comparison with chemotherapy alone.

**Materials and Methods:**

A systematic literature search was performed to identify related citations up to January 31, 2017. Data were extracted, and summary incidence rates, Peto odds ratios (Peto ORs), and 95% confidence intervals (CIs) were calculated.

**Conclusions:**

The use of ALK-TKIs significantly increases the risk of developing high-grade ILD and QTc prolongation in lung cancer patients. Clinicians should pay attention to the risks of severe ILD and QTc prolongation with the administration of these drugs.

## INTRODUCTION

Lung cancer remains the most commonly diagnosed cancer and the leading cause of cancer mortalities globally [1]. The majority of lung cancer (about 85%) are classified as non-small cell lung cancer (NSCLC) [2]. In the past decade, many genomic abnormalities, such as epidermal growth factor receptor (EGFR) mutation and anaplastic lymphoma kinase (ALK) rearrangements, have been identified in NSCLC. And molecular anti-cancer agents targeting these genomic abnormalities provide novel treatment options for these patients [3–6]. It has been found that ALK gene-rearrangements occur in approximately 5% of patients with NSCLC [7]. Crizotinib, an oral small-molecule tyrosine kinase inhibitor against ALK, MET and ROS1 kinases, is the first approved ALK-inhibitor (ALK-i) by Food and Drug Administration (FDA) as first-line treatment for ALK-positive advanced NSCLC [8]. However, acquired resistance to crizotinib by activing alternative signaling pathways usually develops within the first year of treatment. Clearly, the development of effective next-generation ALK inhibitors for the advanced NSCLC is desperately needed. Indeed, two next-generation ALK inhibitors ceritinib and alectinib have shown high activities in either crizotinib-pretreated or –naïve population [9–11]. As a result, the treatment landscape of ALK-positive NSCLC is expected to evolve rapidly.

In comparison with traditional cytotoxic anticancer therapies, ALK-TKIs have been reported with a unique spectrum of adverse events, although the common toxicities of ALK-TKIs, such as nausea and diarrhea, are well tolerated and manageable [4, 12, 13]. Concerns have arisen regarding the incidence and risk of interstitial lung disease (ILD) events and QTc interval prolongation with the use of ALK-TKIs, although the role of ALK-TKIs in the development of these toxicities remains unknown [14]. Current recognition of its risk according to single individual trial, but these studies generally have small sample size and patient selection bias. To our best knowledge, there has been no systematic review to synthesize these data and the overall risk of these toxicities induced by ALK-TKIs hasn’t been defined. Therefore, we conducted a meta-analysis and systematic review of published phase II and III clinical trials to investigate the overall risk of developing ILD and QTc prolongation in NSCLC patients treated with ALK-TKIs.

## RESULTS

### Search results

**Figure 1:**
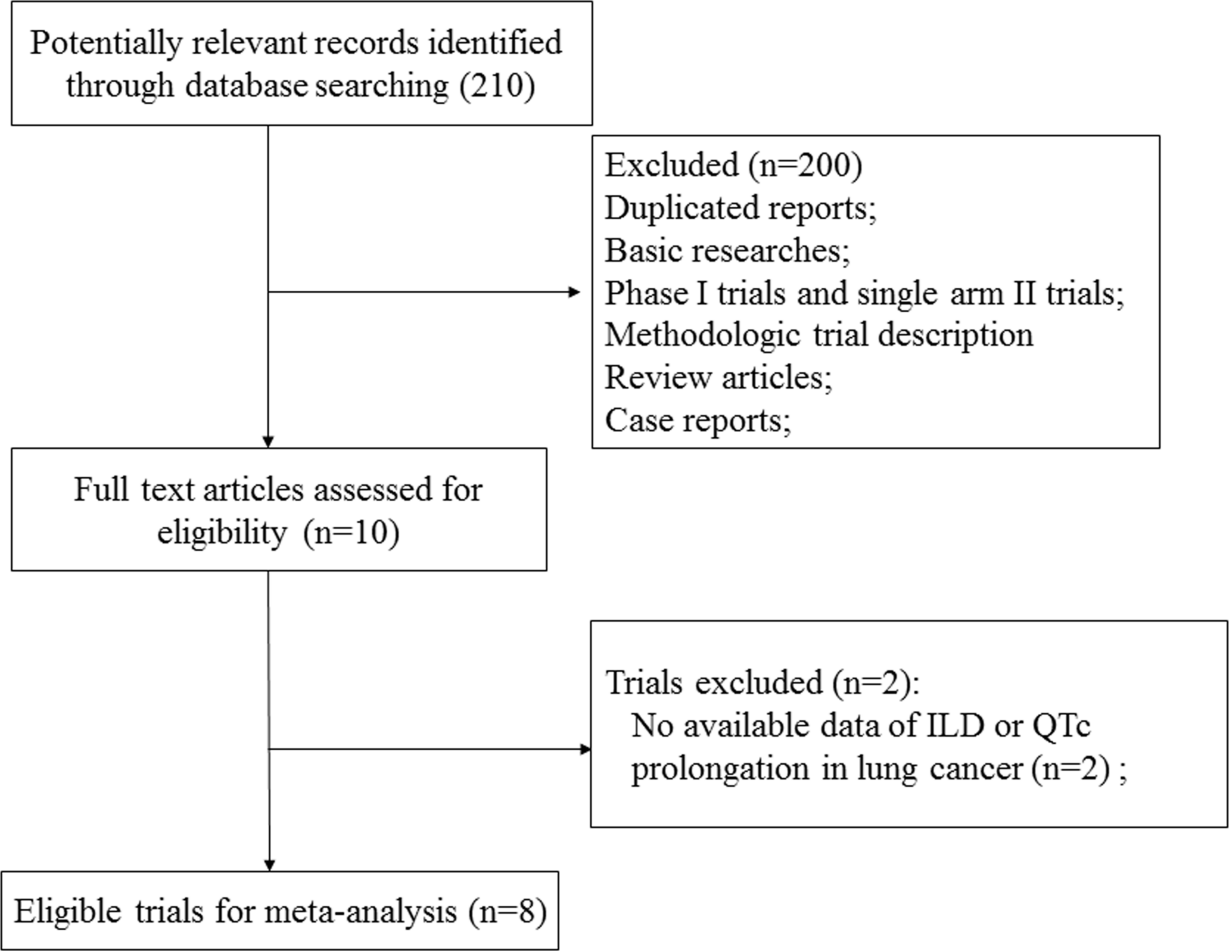
Flow chart of trial selection process in the meta-analysis

We initially found 210 relevant citations of ALK-TKIs in NSCLC patients. After excluding review articles, phase I studies, case reports, editorial, letters, commentaries, meta-analyses and systematic review (Figure 1), we selected 8 prospective trials, included 3 phase III [15–17] and 7 phase II trials [18–22] (Table 1). A total of 1,770 patients were finally included in the present study. Table 1 listed the baseline characteristics of patients and studies. The quality of each included study was roughly assessed according to Jadad scale, and all of the three randomized controlled trials were open-label controlled trials, thus had Jadad score of 3.

**Table 1:** Baseline characteristics of eight included prospective trials

Authors/year	Phase	Patients enrolled	Treatment Arm	Median age (y)	Median PFS (m)	Median OS (m)	No. for analysis
**Kwak E.L. et al/2010**	Expansion cohort	82	Crizotinib 250mg bid po	51	NR	NR	82
**Camidge D.R. et al/2012**	Expansion cohort	149	Crizotinib 250mg bid po	52	9.7	NR	149
**Shaw A.T. et al/2013**	III	347	Crizotinib 500mg bid po	51	7.7	20.3	172
			Chemotherapy	49	3	22.8	171
**Shaw A.T. et al/2014**	Expansion cohort	81	Ceritinib 750mg qd po	53	NR	NR	81
**Solomon B.J. et al/2014**	III	343	Crizotinib 500mg bid po	52	10.9	NR	171
			Chemotherapy	54	7	NR	169
**Shaw A.T. et al/2016**	II	87	Alectinib 600mg bid po	54	NR	NR	87
**Kim D.W. et al/2016**	Expansion cohort	255	Ceritinib 750mg qd po	NR	NR	NR	255
**Soria J.C. et al/2017**	III	376	Ceritinib 750mg qd po	55	16.6	NR	189
			Chemotherapy	54	8.1	NR	175

Abbreviations: PFS, progression-free survival; OS, overall survival; NR, not reported.

### Incidence and risk of ILD with ALK-TKIs

**Figure 2:**
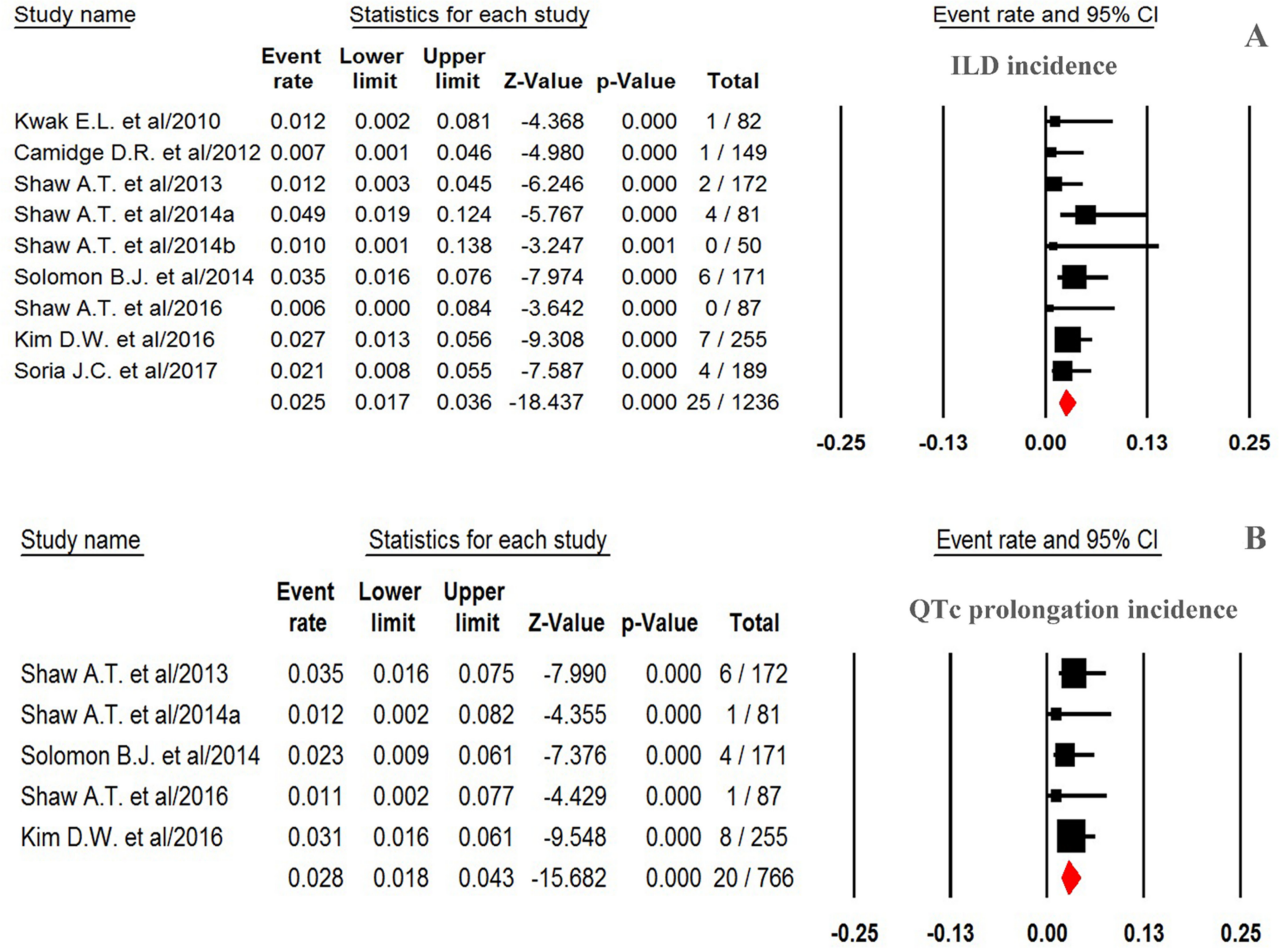
Forest plot for meta-analysis of incidence of high-grade ILD and QTc prolongation in NSCLC patients assigned ALK-TKIs

**Figure 3:**
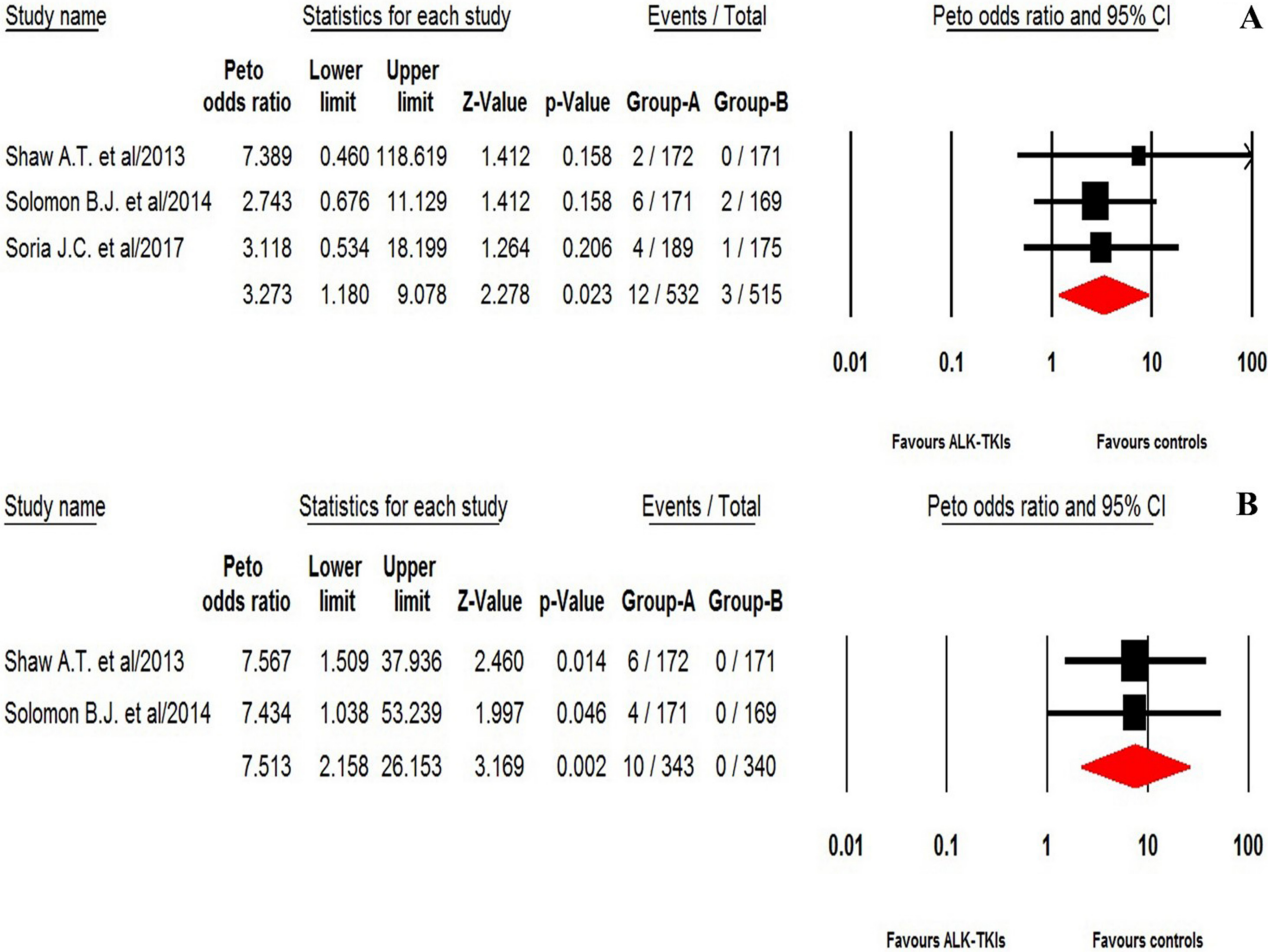
Relative risk of ALK-TKIs-associated high-grade ILD and QTc prolongation from randomized controlled trials

For calculating the overall incidence of grade 3–4 ILD events, a total of 1,236 NSCLC patients were included: the events of ILD was reported in 25 out of 1,236 NSCLC patients received ALK-TKIs with an incidence of 2.5% (95% CI: 1.7–3.6%, Figure 2A). The pooled risk of developing high-grade ILD events was 3.27 (95%CI: 1.18–9.08, *p* = 0.023) in NSCLC patients received ALK-TKIs in comparison with chemotherapy alone (Figure 3A).

### Incidence and risk of QTc prolongation

For calculating overall incidence of grade 3–4 QTc prolongation, there were a total of 766 patients in our analysis: the events of QTc prolongation was reported in 20 out of 766 NSCLC patients receiving ALK-TKIs yielding an overall incidence of 2.8% (95% CI, 1.8–4.3%, Figure 2B). The RR (fixed effect) to develop grade 3–4 QTc prolongation was 7.51 (95% CI, 2.16–26.15; *p* = 0.002, Figure 3B) in NSCLC patients received ALK-TKIs in comparison with controls.

### Publication bias

We did not perform publication bias analysis due to limited randomized controlled trials in the present study.

## DISCUSSION

Recently, as the number of patients receiving new generations of tyrosine kinase inhibitors increase, TKIs associated ILD and QTc prolongation is being more commonly seen in clinical trials. Clinicians pay more and more attention to the risk of pulmonary and cardiac toxicities associated with these drugs. Two previous meta-analyses have found that the use of EGFR-TKIs (gefitinib and erlotinib) in advanced NSCLC significantly increases the risk of developing both all-grade and fatal ILD events [23, 24]. Additionally, increased risk of drug-induced QTc prolongation has been also reported with vascular endothelial growth factor receptor (VEGFR) tyrosine kinase inhibitors (TKIs) [25, 26]. However, to our best knowledge, the overall incidence and risk of ILD and QTc prolongation associated with ALK-TKIs remains undetermined.

The present study has shown that the use of ALK-TKIs are associated with a significantly increased risk of developing high-grade ILD and QTc prolongation. The incidences of high-grade ILD and QTc prolongation is 2.5% (95% CI 1.7–3.6%), and 2.8% (95% CI 1.8–4.3%), respectively. In comparison with chemotherapy alone, the use of ALK-TKIs significantly increases the risk of high-grade ILD (Peto OR, 3.27, 95%CI: 1.18–9.08, *p* = 0.023) and QTc prolongation (Peto OR 7.51, 95% CI, 2.16–26.15; *p* = 0.002), respectively. A total of four patients permanently discontinued as a result of crizotinib-related pneumonitis, and three fatal ILD related with crizotinib were reported in these trials. In addition, seven patients in the ceritinib group had interstitial lung disease, resulting in permanent discontinuation of ceritinib treatment, and two fatal ILD with ceritinib were reported in our trials. The present study would help physicians and patients to fully recognize the overall risk of ILD and QTc prolongation with ALK-TKIs therapy in NSCLC patients. In order to reduce morbidity and mortality with ILD and QTc prolongation during the administration of ALK-TKIs, physicians should clearly recognize these risks and should pay close monitoring to ILD and QTc prolongation in NSCLC patients receiving ALK-TKIs.

Currently, the specific mechanism underlying TKI-induced ILD is undetermined. A previous research found that the EGFR signaling pathway play an important role in impairing lung epithelium [27]. As a result, inhibition of this signaling pathway might lead to EGFR-TKI-induced ILD. However, these is little knowledge about the mechanism of ALK-TKI related ILD. Currently, these are no specific guidelines for the treatment of ALK-TKIs-related ILD because these is lack of studies addressing this issues. The packet insert recommends that patients should be monitored for pulmonary symptoms indicative of ILD, and ALK-TKIs should be permanently discontinued for patients diagnosed with a drug-related ILD. Although systemic corticosteroids are often recommended and administered for drug-induced ILD, an effect of systemic corticosteroid administration on survival is not found. However, the relatively small sample size and retrospective nature of the present review do not allow us to rule out a potential benefit.

Cardiac toxicity by I_Kr_ channel inhibition could be another major liability for some classes of novel targeted drugs. During preclinical drug development, some promising drugs could be abandoned due to the detection of I_Kr_ channel inhibition or *in vivo* QT prolongation. The specific mechanism underlying TKI-related QTc prolongation is thought to be associated with a drug’s three-dimensional molecular structure, which could interact with myocardial hEGR K^+^ channels. In addition, preclinical studies have found that ALK-TKIs could inhibit the ion channel which might be associated with delayed-rectifier K+ current in the heart. Inhibition of the I_Kr_ channel can result in QTc prolongation, which can lead to life-threatening cardiac toxicities [28]. According to the packet insert, ALK-TKIs should be immediately discontinued once drug-induced high-grade QTc prolongation occurs. The subsequent treatment has to be carefully considered if the patient recovery from the QTc prolongation, Additionally, routine EKG monitoring are recommended to be performed in patients receiving ALK-TKIs due to its increased risk of developing severe QTc prolongation.

Our meta-analysis has several limitations. Firstly, ILD is a complex disease encompassing many different pathological diseases. Also, the diagnostic criteria for ILD varies among included studies. Additionally, these studies are conducted at various centers, and the ability to detect ILD events might vary among these institutions, which could have potential bias in reporting incidence rates of ILD. Second, our study is a meta-analysis of published studies, but not a meta-analysis of individual patient data, and confounding factors at the patient level cannot be properly investigated in our study. Finally, publication bias is an important issue in the meta-analysis. We do not perform analysis to detect the publication bias due to limited included study for analysis.

## MATERIALS AND METHODS

### Clinical end point

The following adverse outcomes, including interstitial lung disease, pneumonitis, and interstitial pneumonia, were considered as ILD events and were included for analysis.

As for high-grade QTc prolongation, grade 3 QTc prolongation defined as QTc ≥ 501 ms and grade 4 QTc prolongation were included for analysis in the present study. Adverse events were defined as per version of the National Cancer Institute’s Common Terminology Criteria for Adverse Events criteria because of its use in the selected trials (NCI-CTC, version 3 or 4; http://ctep.cancer.gov).

### Data sources

We conducted an independent review of citations in several databases, including the Pubmed (data from Jan 2000 to Jan 2017), Embase (data from Jan 2000 to Jan 2017) and the Cochrane Library. Key words were “ALK-TKIs”, “ALK inhibitors”, “crizotinib”, “ceritinib”, “alectinib”, “non-small-cell lung cancer”, “non-small-cell lung carcinoma”, “prospective trials”, “interstitial lung disease”, “pneumonitis”, “interstitial pneumonia”, and “QTc prolongation”. The search was limited to prospective clinical trials published in English. We also searched abstracts containing the term “ALK-TKIs” that were presented at annual meetings to identify relevant studies, including the American Society of Clinical Oncology (ASCO) and European Society of Medical Oncology (ESMO). Additionally, we searched the clinical trial registration website (http://www.ClinicalTrials.gov) to obtain information on the registered prospective trials. Each publication was reviewed and in cases of duplicate publication only the most complete, recent, and updated report of the clinical trial was included in the meta-analysis.

### Study selection

Phase I trials were excluded because of inter study variability in drug dosing and the limited sample sizes, and only prospective phase II/III trials evaluating ALK-TKIs in NSCLC patients with adequate data on toxicities of special interest were incorporated in the analysis. Study selection was conducted according the Preferred Reporting Items for Systematic Reviews and Meta-Analyses (PRISMA) statement. Clinical trials that met the following criteria were included: (1) prospective phase II or III trials involving NSCLC patients; and (2) available data regarding events or incidence of ILD events/QTc prolongation and sample size. If multiple publications of the same trial were retrieved or if there was a case mix between publications, only the most recent publication (and the most informative) was included.

### Data extraction

Two independent investigators conducted the data abstraction, and any discrepancy between the reviewers was resolved by consensus. The following information was extracted for each study: first author’s name, year of publication, trial phase, number of enrolled subjects, treatment arms, number of patients in treatment and controlled groups, median age, median progression-free survival, and adverse outcomes of interest (high-grade ILD and QTc prolongation).

### Statistical analysis

The primary summary measures were incidence, Peto odds ratios (ORs), and corresponding 95%CIs. All statistical analyses were performed by using Version 2 of the Comprehensive MetaAnalysis program (Biostat, Englewood, NJ). For the calculation of incidence, the number of patients with high-grade ILD and QTc prolongation in ALK-TKIs group and the total number of patients receiving ALK-TKIs were extracted; the proportion of patients with high-grade ILD and QTc prolongation and 95% confidence interval (CI) were derived for each study. To calculate Peto ORs, patients assigned to ALK-TKIs were compared only with those assigned to control treatment in the same trial. We used the Peto method to calculate odds ratio (ORs) and 95%CI confidence intervals (CIs) because this method provided the best confidence interval coverage when dealing with low event rates [29]. Between-study heterogeneity was estimated using the χ^2^-based Q statistic [30]. The *I*^2^ statistic was also calculated to evaluate the extent of variability attributable to statistical heterogeneity between trials. A statistical test with a *p*-value less than 0.05 was considered significant. Study quality was assessed by using the Jadad scale based on the reporting of the studies’ methods and results [31].

## CONCLUSIONS

In summary, the present study has found that the use of ALK-TKIs significantly increases the risk of developing high-grade ILD and QTc prolongation. Clinicians should be aware of the risks and benefits with ALK-TKIs treatment in NSCLC patients. Finally, further research are still needed to clearly investigate the predictive factors of these toxicities with administration of ALK-TKIs treatment.

## Abbreviations

ALK: anaplastic lymphoma kinase
TKIs: tyrosine kinase inhibitors
NSCLC: non-small cell lung cancer
ILD: Interstitial lung disease
Peto ORs: Peto odds ratios
CIs: confidence intervals
FDA: Food and Drug Administration
VEGFR: vascular endothelial growth factor receptor
